# Dopaminergic Challenge With Bromocriptine in Patients With Severe Brain Injury

**DOI:** 10.1186/2197-425X-3-S1-A485

**Published:** 2015-10-01

**Authors:** JB Celik, A Duman, O Arun, IO Onal, O Ilban, AE Sonmez

**Affiliations:** Selcuk University Medicine Faculty, Anesthesiology and Reanimation, Konya, Turkey

## Introduction

Bromocriptine Mesylate (BC) is an ergot derivative with potent dopamine receptor agonist activity. It is licensed to reduce plasma levels of prolactin. BC has central nervous effects, and is used in patients with Parkinson's disease. There are few randomized controlled trials with BC conducted in moderate brain injury (BI) with conflicting results (1). We aim to present our single center experience on dopaminergic challenge using off-label BC in patients with severe BI.

## Methods

Glasgow Coma Score (GCS) are 3-10 (GCS: worst score = 3, best score = 15) at beginning of BC administration. Patients received 2.5mg of BC q6h after hemodynamic stability was ensured and no further neurologic improvement was observed during course of management. BC was started and discontinued on the discretion of the ICU team. Long term cognitive tests are under assessment.

## Results

A total of 18 patients were treated with BC. the average age was 48.9 (82-18) (12M/6F).

## Conclusions

Our results show some neurologic improvement as assessed by GCS. More research is warranted before BC can be recommended in BI.

**Table 1:** GCS of patients on admission, beginning treatment, end of treatment and discharge. GCS of patients on admission, beginning treatment.

Patient Number	GCS admission	GCS start	GCS end	BC started on day	Days on BC	Days in ICU	GCS discharge
1	3	4	8	29	9	38	8
2	5	5	5	16	8	39	5
3	15	10	14	25	4	29	14
4	3	3	6	11	30	64	12
5	3	3	5	6	23	39	5
6	3	6	10	28	8	70	died
7	3	3	4	9	7	75	6
8	3	4	4	24	15	40	died
9	4	4	5	9	34	49	8
10	4	4	7	10	35	46	died
11	3	8	3	9	7	18	died
12	3	4	4	11	7	18	8
13	3	6	6	11	19	30	6
14	6	6	8	1	21	37	10
15	5	5	5	9	4	51	8
16	5	6	7	11	30	47	7
17	3	6	5	28	25	64	died
18	3	4	5	32	17	59	5
